# Risks to Early Childhood Health and Development in the Postconflict Transition of Northern Uganda

**DOI:** 10.1155/2012/820290

**Published:** 2012-02-16

**Authors:** Theresa A. McElroy, Stella Atim, Charles P. Larson, Robert W. Armstrong

**Affiliations:** ^1^Canadian Child Health Clinician Scientist Training Program, and University of British Columbia, Vancouver, BC, Canada V6T 1Z1; ^2^Child Health and Development Centre, Makerere University, Kampala, Uganda; ^3^Centre for International Child Health, BC Childrens Hospital and University of British Columbia, Vancouver, BC, Canada V6T 1Z3; ^4^Medical College, East Africa, Aga Khan University Nairobi, Kenya

## Abstract

Research from numerous fields of science has documented the critical importance of nurturing environments in shaping young children's future health and development. We studied the environments of early childhood (birth to 3 years) during postconflict, postdisplacement transition in northern Uganda. The aim was to better understand perceived needs and risks in order to recommend targeted policy and interventions. *Methods*. Applied ethnography (interview, focus group discussion, case study, observational methods, document review) in 3 sites over 1 year. *Results*. Transition was a prolonged and deeply challenging phase for families. Young children were exposed to a myriad of risk factors. Participants recognized risks as potential barriers to positive long-term life outcomes for children and society but circumstances generally rendered them unable to make substantive changes. *Conclusions*. Support structures were inadequate to protect the health and development of children during the transitional period placing infants and young children at risk. Specific policy and practice guidelines are required that focus on protecting hard-to-reach, vulnerable, children during what can be prolonged and extremely difficult periods of transition.

## 1. Introduction

Research from biology to the social sciences continues to build strong evidence demonstrating that the conditions in which young children live and grow are determinants of their health and developmental trajectories [1–13]. The World Health Organization's Commission on Social Determinants in Health [14, 15] commissioned a report on early childhood development, completed in 2007 [2, 3]. Its key messages include the following: the early years are the most critical period in human development; numerous physical health, mental health, and social outcomes are strongly influenced by conditions experienced in early childhood; failure to provide nurturing environments not only impacts individual life chances but can threaten sustainable, peaceful, equitable development in society; caregivers require support from government and other agencies (local to international) to create nurturing environments for children [2]. Stimulation received in responsive and nurturing relationships, exposure to safe environments, and meeting of physical needs like adequate nutrition [1] provide what children need for healthy development.

All too often young children are exposed to conditions that do not meet their most basic needs for health and development. The literature conservatively estimates that worldwide more than 200 million children under the age of 5 years living in low income countries (such as Uganda) fail to reach their developmental potential [6]. In such contexts research has demonstrated that children are faced with numerous factors associated with significant risk to development including poverty, malnutrition, poor health (preventable and treatable infectious diseases), and psychosocial factors (exposure to violence, maternal depression, lack of stimulation/social interaction in the home environment) [12]. Those living in the least nurturing environments and with the greatest levels of risk manifest the poorest outcomes. One such situation is that of prolonged war and internal displacement.

War and displacement create conditions of high risk or vulnerability for children's development and well-being through increased exposure to conditions such as economic devastation, destruction of basic infrastructure (i.e., health care, education, legal protection), reductions in hygiene and sanitation with resultant elevations in disease spread, and breakdown of social support structures that are thwarted from nurturing and protecting children [16–19]. These conditions of ubiquitous collapse of nurturing environments do not end with the cessation of conflict but have enduring impact on children, families, communities, and societies; impact that requires time and prolonged effort to rebuild and restore [17, 19]. Additionally, factors such as lost infrastructure and mass population movement can make children difficult to reach and hence difficult to serve with appropriate interventions to mitigate risks. Nonetheless, the postconflict transition can be of long duration and the negative impact may be the most challenging to children in the first three years of life when critical and sensitive periods of brain development can be compromised. Accordingly, this study sought to document the environments of early childhood (birth to 3 years) in a postconflict, postdisplacement setting in northern Uganda in order to better understand the needs and risks to health and development and to examine ways of targeting policies and practices that can serve to mitigate risk in these difficult circumstances.

## 2. Methods

### 2.1. Background. The Region of Northern Uganda

The 20-year war waged in northern Uganda (1986–2006) predominantly between the LRA rebel insurgents and the government forces was politically complex, enduring, and vicious. Numerous authors have written detailed accounts of its history, politics, and nuances [20–24]. From the earliest days of the war, civilians were subject to looting and/or destruction of their familial resources such as cattle and livestock, devastation of health and educational infrastructure, and personal violence—rapes, beatings, killings, and mass abductions to populate the rebel forces. This included an estimated 66,000 youth ages 14 to 30 [25].

In 1996, the Ugandan government formally established “protected villages” (internal displacement camps) in urban or central gathering areas throughout the north. Many people fled to such camps, but many more were forcibly displaced into them. Despite the promised protection from violence and destruction that the camps were meant to provide, atrocities continued against the civilian population perpetrated by a range of actors [21, 24, 26, 27]. In addition, camps were frequently reported to: lack services and infrastructure which contributed to food insecurity and poor health, have serious overcrowding and unhygienic conditions leading to disease spread, and create conditions of severe poverty and inability to maintain independent livelihood [21, 24, 26, 28–30]. A health and mortality survey conducted in these camps in 2005 suggested that under 5 mortality (U5MR) rates were well above the emergency thresholds (i.e., 2.31 deaths per 10,000 children at risk per day), with the leading causes of child death being malaria/fever and a disease constellation that included diarrhoea, thrush, and malnutrition [29]. Relief agency involvement increased dramatically from the time camps were established [31 in 21] to meet the emergency needs of a rapidly growing displaced population, which reached approximately 2 million or 90% of the regional population by the end of 2005 [29].

The conflict in northern Uganda ended in 2006 with the signing and renewal of a cessation of hostilities agreement [21]. The process of repatriation and reconstruction, which was still continuing in 2009-2010 when this research was conducted, was complex, arduous, and protracted.

### 2.2. Population and Site Selection

The source population was Acholi people (the dominant ethnic group in the region) living in the Amuru district of northern Uganda. This district was chosen as it was a relatively newly created district (2005) and according to key informants was underresourced and underserved compared to neighbouring districts. It was also reported to be a region greatly impacted by war. In 2005 the district held the highest number of displacement camps in the Acholi region with 34 camps housing a population of approximately 257,000 [31]. At the start of the study in May 2009, a reported 37% of people still remained living in the camps in Amuru [32], and, by the end in June 2010, many of the camps had been officially closed with UNHCR reporting only 14% of the internally displaced population remained in camps and settlements [31]. The study captured the period of movement for 23% of the population in this district.

The target population in this study was children birth to three years old, from rural Agrarian families (the prewar livelihood and lifestyle of the majority of the population in the region) who experienced conditions of conflict and displacement. As this age group was highly dependent and unable to speak on their own behalf, the team used a mix of direct observation of children in their natural contexts and proxy respondents-family caregivers and community members directly connected to young children.

The study population came from villages and internal displacement camps within three subcounties geographically spaced throughout the Amuru District: Pabo, Amuru, and Anaka. The villages and camps were selected purposively from the source population with the guidance of key informants to reflect a range of remoteness/accessibility, resource availability, and stage of resettlement/repatriation of the population (summarized in Table 1).

### 2.3. Participant Selection

The sampling was purposive or criterion based. Participants were chosen due to their possession of the characteristics that allowed for in-depth exploration of the topic of study [33]. There were two key inclusion criteria for these focus group and interview participants: (1) experience of conflict and displacement and (2) knowledge or experience of young children as direct caregivers, community leaders working for children and/or people with decision-making power and/or influence in regards to the well-being of young children in the northern Ugandan context. Within these broad criteria, a heterogeneous sample (also known as maximum variation sampling) was sought to capture the breadth of experiences and maximize the potential for identification of all factors associated with the topic of study [33]. A total of 162 people were either interviewed or participated in one of 26 focus group discussions (FGD): 35 primary female caregivers, 25 fathers, 22 elders, 29 sibling caregivers, and 51 leaders, both formal (teachers, health workers, government officials, NGO workers, village health teams, child protection committee members) and traditional (traditional chiefs, traditional birth attendants). There were numerous other leaders and community members who were engaged informally in dialogues during research introductions or during other activities where participant observation was conducted such as when the team was referring children to services.  Table 2 provides an overview showing the diversity of participants. Furthermore, in all sites people with disability were included, either a caregiver or the young child who was the focus of observation, making up approximately 10% of the families in this study. Impairments experienced by these individuals included quadriplegia, amputation (from landmine), visual impairment, impaired mobility (i.e., high muscle tone from neurological insult-cerebral malaria), hearing impairment (from chronic untreated ear infections).

Local leaders assisted with identifying participants within their communities according to the identified criteria. On occasion, participants referred other participants, and/or the team directly approached caregivers who had young children. This variation in recruitment helped to guard against biases that could have arisen from having participants identified solely through leadership [34].

### 2.4. Conduct of Study

The research project had an interdisciplinary advisory committee composed of both Ugandan and Canadian professionals from the fields of paediatrics, preventative medicine and public health, anthropology, population health, and occupational therapy. The core fieldwork team consisted of an experienced ethnic Acholi researcher, offering the emic perspective (cultural insider) and a Canadian researcher with a background in paediatric occupational therapy, international health and qualitative research offering the etic perspective (cultural outsider). Two Acholi research assistants, one male and one female assisted with data collection on occasion and worked on transcriptions/translations and team discussions of emergent themes. Training and preparation of the core fieldwork team occurred over a one-month period. The diversity of the research team provided both investigator and theoretical triangulation. Triangulation is a concept applied in qualitative research whereby the topic of interest is approached from a number of different angles (researchers, theoretical basis, methods, sites) to offset weaknesses, challenge biases, confirm the validity of findings, and, deepen understanding of concepts [35].

### 2.5. Data Collection Methods

Data collection occurred over a 1-year period, June 2009 to June 2010. Both the Acholi and the Canadian researcher jointly conducted the majority of interviews, FGD, and observations. Dialogue occurred in the local language of Lwo for all except two leader interviews and was digitally audio recorded and then transcribed and translated into English. During development of the question guides, a rigorous translation process was applied to ensure concepts and meanings remained consistent from English to Lwo [36].

This study applied multiple data collection methods to strengthen the depth, authenticity, and credibility of the study through triangulation of data sources [35, 37]. Under the methodological approach of applied ethnography, seven data gathering methods were used.

#### 2.5.1. Semistructured Interviews and Focus Group Discussions

Interviews conducted with individuals lasted approximately one hour. Focus group discussions (averaging 4–6 people) lasted approximately 2 hours (ranged from 35 minutes with sibling caregivers to 5 hours over 2 sessions with elder women). The open-ended semistructured interviews and FGDs were formulated to explore the context of children's lives and elicit perceptions about barriers and facilitators to young children's health and development; questions and probes evolved over the duration of the study to fully explore new ideas as they emerged. Participants were asked a range of questions to bring forth their knowledge, attitudes, beliefs, practices, and perceptions about life circumstances/environments of young children (sample of guiding questions in Table 3). Interviews and FGD were conducted in a range of locations such as homes (inside or outdoors under a tree), community buildings such as churches, or places of work (for leaders); participants decided on the location and whether they wanted to complete the interview/FGD in one session or multiple sessions. Privacy was ensured in all settings. Following interviews with primary female caregivers, weight and height data was collected for the youngest child under 3 years as an objective source of data to corroborate caregiver's perceptions about nutritional status.

#### 2.5.2. Case Studies

This method involved visiting 6 children and their families every 4 to 6 weeks for a duration of 6 months to 1 year as they transitioned following conflict.

#### 2.5.3. Participant Observation in Camps, Villages, and in Service Settings

The two primary field researchers (Canadian and Acholi) observed young children (with parental permission) and recorded what they were doing, how they were doing it, with whom, and where in their daily lives and natural settings. The observations were primarily recorded by the Canadian researcher in field journals and were confirmed with the Acholi researcher. Additionally, living and working in the research sites afforded numerous opportunities for recording observations of informal interactions that shed light on the context of young children's lives, a technique called participant observation. For instance, on several occasions the research team brought children and caregivers to services such as hospital and recorded prominent features of these experiences. As Good and Thorogood (2009) explain, “Observational methods allow the researcher to record the mundane and unremarkable (to participants) features of everyday life that interviewees might not feel were worth commenting on and the context within which they occur” (p. 148).

#### 2.5.4. Document Analysis

This method involved reviewing governmental and nongovernmental reports, meeting minutes, and news stories for information relevant to child-focused services provided or lacking.

The two field researchers held debriefing meetings each day on the journey home from the research site where observations, emerging themes, clarifications, meanings, and areas for further exploration were discussed. Field notes on impressions, observations, challenges, and reflections were taken by both researchers and were kept together in field diaries.

### 2.6. Ethics

Ethical approval was obtained through the Makerere University (Uganda), The Uganda National Council of Science and Technology, and the University of British Columbia (Canada). Additionally, permissions were sought from senior leadership at the district and subcounty levels and from local leadership in camps and villages, that is, the local council members in the region and/or the Rwot Kweri (traditional leaders). Informed consent was received from all participants. Consent forms were read aloud to participants in the local language to ensure that literacy and language were not barriers to providing consent. Confidentiality was maintained by removing identifiers from responses in reports and publications, and participants chose the location for their interview or FGD.

### 2.7. Analysis

Content analysis was used to identify themes to describe the environment and the local perceptions of its influence on early childhood health and development. Data analysis was an iterative, on-going process from the beginning of data collection. Accordingly, the data collection guides used for probing were revised numerous times over the course of the year as analysis revealed new themes requiring further exploration as well as themes that had reached saturation. During fieldwork, a team approach was employed in the analytical process to ensure thoroughness through alternative interpretations [38] and involved both members of the fieldwork team and the advisory committee.

At the end of data collection, all transcripts were imported into qualitative analysis software to assist with organizing and sorting the data (field notes were analysed manually as these were handwritten). The transcripts alone consisted of over 1700 single-spaced typed pages and therefore required organization into manageable units. The initial organizational framework applied to the raw data was to delineate it by research objectives. Using the analysis software, sections of the transcripts were highlighted and then assigned a label or code, for instance, all data related to postconflict transition were coded “transition.” Once all of the transcripts were coded, the software program generated a report of raw data for each objective. The data set generated for early childhood in postconflict transition was the source of this paper.

Once there was a dataset generated for each objective, thematic analysis was applied. This involved noting that “certain phrases, events, activities, behaviours, ideas or other phenomena occur repeatedly in the data” [39, p. 46] and devising a code (or label) for these segments of the data according to their message or what they were about. For instance “socioeconomic environment” was used to code statements about poverty, household resources, crop failures, food aid, and so forth. Once again, the software generated reports of raw data excerpts delineated by these broad themes. The ideas under each theme were then summarized. The team compared and contrasted between and within participant's reports, categories and typologies were generated, and dialogue ensued about meaning of the data [35]. Attention was given to both information that repeated regularly and information that was unusual or presented alternate perspectives. Furthermore, patterns and relationships in the data were also sought. For instance, the data on “socioeconomic environment” was examined across participants in different phases of postconflict transition, from those still living in camps to those who had been settled in villages for varying lengths of time. Doing this allowed for exploration of associations between resettlement, resources, and reports of children's status. This process was completed in consultation with the advisory committee and reviewed by the fieldwork team to ensure agreement on results of the analysis.

## 3. Findings

An estimated 90% of the northern population lived in displacement by the end of war, and consequently, the majority of children in the region were born and raised in camps. While issues of innumeracy made it difficult to quantify the exact number of years participants spent in displacement, most reported living in camps for more than a decade. After such a prolonged period of restricted living, the move back to rural agrarian villages was a major transition that took time and impacted all facets of life; still, it was welcomed. All participants indicated a desire to get children out of camps and away from the numerous threats to their health, safety and well-being. These included overcrowded conditions where disease spread readily, the undernutrition arising from lost livelihood, and the uncontrolled exposure to negative social influences such as violence and alcoholism.

This research purposefully sampled families in various phases of postconflict transition in order to capture and understand the experiences and environments of young children across resettlement. The findings have been grouped into four sections which will outline broad themes corresponding to children's environments during transition: (1) the physical environment, (2) the socioeconomic environment, (3) the social (care-giving) environment, (4) the regional resource and service environments.

### 3.1. The Physical Environment and Process of Transition

This section highlights themes relating to the physical process of transition that occurred following conflict end when families began to move back to rural villages. The major subthemes in this data were (1) the caregivers' focus on physical rebuilding of home and livelihood and (2) vulnerable families without land and social support.

#### 3.1.1. Focus on Rebuilding

After prolonged displacement, the move back to former homesteads was reported to be deeply challenging for caregivers. Rural village homes traditionally constructed of baked bricks and grass thatch roofs had long since disintegrated and had to be rebuilt from nothing along with structures like bathing shelters, latrines, and wells. For many, the agricultural lands had gone fallow, which meant significant periods of time, and heavy and prolonged labour was required to clear, plant, and reestablish crops needed to feed the family. Furthermore, belongings, while usually minimal, had to be transported gradually from the camps, usually carried on people's heads or, less frequently, with a bicycle or hired motorbike. These gruelling activities of resettlement had to be prioritized in order to re-build homes and livelihoods. However, while caregivers worked, young children could not live in remote and wild regions without shelter, food, and services such as health care. Consequently, both caregivers and leaders reported that children were being left in camps while adult family members moved back and forth to the village to complete the necessary work (discussed further in social-cultural environment section).

I am living in the camp but not fully. I have one leg here and one leg in the village…. We have homes (villages) which are very far, some of our homes are more than 10 miles away from here and you cannot gather your things all at the same time to return to the village. I am picking my things one by one and if I was to go at once, life may not be easy for me there because I have just cleared my gardens (agricultural land). My children may lack food to eat when I take them there so I have been leaving my children behind. My wife, and I we have been going back home to build our huts and to clear gardens and to get a proper water source for drinking. With the house also I have just made the bricks, I do not only need one hut, I need many huts because I have children. I have only planted potatoes there. When I have planted enough crops, and they are ready for harvest, that is when I will be fully in my village, but right now I am not yet ready. Some of the crops I planted were destroyed by sunshine. *Father in FGD held in a camp. *

#### 3.1.2. Without Land and Support

While most families encountered in camps were in the process of rebuilding and resettling, in each camp studied, there were families who were unable to return to their homes and agricultural lands despite their desire to do so. In Acholi culture, married women moved to their husband's land and males possessed many of the skills essential to building homesteads such as constructing structures and clearing fields. Consequently, female or child headed households, elders, people with disabilities or weaknesses, and other categories of caregivers who had limited social support (because male relatives had died, abandoned, were abducted, or lived distantly), often spoke of being without return options. This included being without access to land that would traditionally have housed and fed their families and/or having no one to help them rebuild. Left in the limbo of camps that were emptying and closing, they spoke of great strain in trying to meet their children's most basic needs.

… It must be the one (the person) who has nobody to help them build a hut who has remained in the camp. The owners of these lands (camps) also want their land to be vacated but now you will find that people have nowhere to go and you are quite confused…. *29-year-old separated mother of 4, still living in displacement camp. *

Well, for me, this war has brought me loss. My husband died, the father of my children. I have remained with children, with no one to help them. Even I do not have any means of taking care of them, it has become very difficult; sorrow almost killed me. I would keep on remaining silent and I became sick. My chest would pain me. There is no one who helps me with the children. Even trying to get a means of living is extremely difficult. *~40-year-old widowed mother of 5, still living in a camp. *

Furthermore, in each camp families were encountered who attempted to begin the process of resettlement only to be stopped by land disputes with kin. Such disputes drove them away from land they felt they had claim to and left them with limited means of providing for young children in their care. These disputes also caused rifts between kin who in the past would have been a source of social support for children providing assistance such as childcare, advice, and/or material resources.

… The conflicts over land are so intense. People are coming back alright, but they have become enemies. People are coming back but there are too many; they ran away from the war, they went and multiplied so much, they came back with their young children. … It is because of land that people have become enemies. It is as if they are no longer relatives…. One family; they disputed over land; they beat each other; they fought with hoes when they were digging in the garden; even with spears…. *20-year-old mother, living with her husband and 4 children (2 biological, 2 of kin) in their return to village. *

### 3.2. The Socioeconomic Environment of Children after Conflict

This section illustrates themes relating to the socioeconomic environment of young children during postconflict transition. The major subthemes are (1) the cessation of wartime subsistence, (2) the origins and experience of poverty after war, (3) young children's unmet needs, (4) hard work, poverty reduction approaches, and persistence for a better future.

#### 3.2.1. The Cessation of Wartime Subsistence

In camps, provisions provided by governmental, nongovernmental, and international organizations had attempted to meet children's basic needs (reports suggested major gaps, but it is beyond the scope of this paper). However, when war ended and there was a call to return home, a substantial portion of war-time subsistence ceased. Many of the provisions that had been supplied to some extent in camps such as food, basic household necessities, and clean water were stopped or reduced to particular populations. This increased vulnerability of children whose families had not yet made a transition from economic and nutritional dependence to independence. When aid stopped, independence had be gained rapidly and almost exclusively through pursuit of productive occupations (primarily agricultural pursuits). Yet as the previous section described, the resumption of livelihoods was not quick or easy.

It is hard to get food for my family because it is hardly there. Because I do not have a garden (agricultural lands), it is hard to get it. I have to do piecemeal work or hired labour in other peoples' garden if I have to eat, if I do not do paid labour, we have to go hungry—with nothing to eat…. The most difficult is this year; it is too much for me. At least last year, people were in the camps we were doing hired labour. We were getting food to eat from them (aid agencies), but now, they are no longer there at all. For us who have remained (in camps), there is no food to eat; this is the worst year of all. —*Single mother (one time widowed, one time separated) caring for 11 children while remaining living in a camp with her elderly mother. *

#### 3.2.2. The Origins and Experience of Poverty after War

Poverty was noted to be oppressive to families and to bring suffering and misery to children. Participants talked of having lost most if not all of their personal wealth and resources (i.e., livestock, crops, structures) during war and displacement to looting, destruction, and disintegration over time. This position of abject poverty was people's starting point in rebuilding their lives and caring for their children.

All of our cattle and the oxen for ploughing were all raided during the war. We had to run bare-handed into the camps with our hands hanging loose by our sides, empty. And when coming back (to resettle in villages) we came back with our hands hanging loose with nothing and although we came back empty handed, without anything, we are struggling at least so that we can carefully nurture our young children to move on ahead; to ensure that they live a better life in the future. —*45-year-old traditional birth attendant, widowed and caring for 5 children. *

With human beings, if they are given birth to at least there should be some training and caring and providing what is necessary for them …. There is no proper food; you are just trying to revive your home. Other varieties of things needed for the compound like cattle, they are not there. Goats are not there … all of these things have defeated the Acholi, they are not there, they are only trying to restore them and this thing is very hard. You have to dig (cultivate), you sometimes eat and at times you do not. Somebody else may come and take your own things from the garden (steal) and this upsets us so much. My words end there. —*34-year-old father of 6, with 2 spouses returning to the village after 19 years of displacement. *

Because agriculture was the primary productive skill base of the majority of people, they were highly vulnerable to weather disturbances and pests that impeded crop growth. In all three sites visited during the study year, droughts and rains caused crops to fail and all participants talked of a period of great and devastating hunger; several referred to this experience as another war. In the past granaries would be filled with food, livestock could be sold, or relatives with plenty could share, but current poverty meant they did not have the resources to see them through such times and their children suffered.

We are in the period of hunger, there is great hunger. The crops planted, sunshine has spoilt it. No food has yet been harvested. That is why we still do not have enough. Life is difficult, the children are not getting enough…. Here children are sent to the hospital, they come back when their health has improved, but when they have been home for a few weeks, because of the bad food they are again taken back to the hospital… — Participant* in FGD of elder woman conducted during drought. *

… You may be interested and willing to see your child grow well but poverty will oppress you. Right now, most people cannot afford to eat a meal in a day, they would wish to see their children grow well, but they lack anything to give their children because there is no food. People's food has dried up in their gardens because of the sunshine (drought). —*Child Protection Committee Member FGD. *

Other factors reported to contribute to poverty subsequent to conflict were caregiver disability or injury sustained during war that impeded the ability to do physical work and provide for children, high fertility rates and large numbers of orphaned children that had to be cared for by extended family, and not having social support and/or land. The more of these factors being experienced, the more difficult it became to generate sufficient income to meet children's needs.

#### 3.2.3. Children's Basic Needs Go Unmet

In the environment of poverty, reports of children's unmet needs were pervasive across all categories of participants during transition. The situation was glaringly visible to the research team, who saw far too many children in camps listless and wasted. The young children who were the subjects of caregiver interviews/observation were weighed as an indicator of nutritional status. Of the 12 children weighed during the drought (May–August), only one 2-month-old, breastfed infant was average weight. Nine of the children were severely malnourished; at or below the first percentile for weight-for-age according to the World Health organization standards. Caregivers made an effort to protect their youngest members during the hunger, with all participants reporting that when food was scarce, young children were prioritized. Yet despite their efforts, often during this period food was simply not available.

… It really gives me heartache. I went to the hospital recently and many children were brought from Pabo; many were blood transfused, many were brought in with swollen bodies, I saw many being blood transfused …… women are trying to take care of children but are failing because their strength and ability is almost gone. They cannot get enough to feed the children and if children are all the time malnourished, they will not grow well and if they do not grow well, in the future there is nothing much that will come out of them… We all here have knowledge about feeding children, but what can knowledge do without food? NRC (Norwegian Refugee Council) taught us how to feed children and even many other NGOs taught us about nutrition and right now there are people who come from UWMFO (Uganda Women and Men's Financial Organization) and they are also teaching us about nutrition, they teach us about agriculture and how we can make children grow up healthy, they have a lot of knowledge they have given us, but what can that do without food?…. In the camp here as you move around, you will find children moving aimlessly, they pick up anything and eat it, even when it is dirty, they pick it up an eat it. When they find a little food thrown somewhere, they fight over it and you will find them crying. Also, there are children here who are not strong against hunger, you will get them crying the whole day. The mother comes back in the evening, cooks at night and the child will eat now while sleepy and that is why many children's bodies are just swelling, it is painful to see the suffering children here. —*Traditional birth attendant FGD, Pabo camp. *

There were frequent reports of children being continuously ill with conditions such as malaria, diarrhoea, skin rash, cough. For example, a child in one of the family case studies was ill 11 of the 15 times we visited her and was admitted to hospital four of those times. While not as severe, the same cycle of illness played out in the other five case studies. Furthermore, all reported times when poverty stopped them from being able to seek medical care, either they could not afford drugs, the cost of transport to the hospital, or the work time that would be lost if children were admitted. Food was the frequently unattainable priority for impoverished caregivers and meeting other needs had to be prioritized by survival ranking.

… Most people still do not have money in their hands, that is why you find children without clothes on their body…. Most of the people in the community, their worry right now is to look for food…. Other things do not matter. They will not mind whether a child has clothing or is sick …. the majority cannot send their children to school, their priority is first food because you as a parent, food is always first for your family, food is the first necessity in a family, no one can do anything without food and most families only have the strength to look for food but not any other thing, they are interested (in promoting children's development) but are just defeated because they do not have any means of survival. —*FGD with the Child Protection Committee, while the group was held in a camp, most had returned to home villages. *

#### 3.2.4. Hard Work, Poverty Reduction Approaches, and Persistence for a Better Future

People spoke of working long hard hours to rebuild, provide, and bring their children home. This hard work contributed to familial resilience and when the drought passed, some participants recounted how their crops began to yield adequately if not abundantly. Children of families who had been settled for longer periods were observed to be healthier and had more appropriate weight-for-age. 

Those who did not have land and social support spoke of long gruelling hours doing casual agricultural labour for cash or food, gathering wild vegetables from the bush, and/or doing small-scale trading for their children's survival. Participants accounts suggest they valued a strong work ethic as the way to get proper food, meet basic needs, and send children for education for a better future.

Now that we have come back to the village, the people are really struggling [working with all their strength] to take care of the children. So their life is really on cultivation, serious cultivation [of their land] and this struggle is aimed at helping so that the children can have the strength in their bodies. So we are actually struggling now that we have come back to the village, on our own land. We want to obtain enough food so that we can get money, then we get other things that can help us put clothes on their bodies and even send them to school to protect the children and this struggle is at a very high degree; it is not decreasing either. —*49-year-old traditional birth attendant, widowed and caring for 6 children. *

The data also revealed a range of additional techniques caregivers used to combat food insecurity, poverty and lack: working communally in groups and rotating through each member's land; exchanging food to gain greater variety; selling harvest to meet other needs; participating in group savings and loans schemes (a skill learned while living in camps). All of these strategies rendered caregivers better able to feed and provide for their children.

### 3.3. Social (Care-Giving) Environment of Children after Conflict

This section presents themes that illustrate the social environment in which young children lived during transition. The major subthemes are (1) survival-based neglect of children, (2) the vulnerability of children without parental care, (3) the social protection of villages.

#### 3.3.1. Survival-Based Neglect

When caregivers had to work daily to meet basic survival needs, preambulatory breastfed babies were generally taken along to the agricultural land where they would remain on the backs of their mothers or be placed in the shade, sometimes with an older child who was brought along to provide care. However, many participants reported that once children were walking they could not be brought along and would be left in the camps. As one Amuru Government official noted “mothers are feeling conflicted because they have to go and work in the fields and if the child is a little bit older, they cannot take them.”

For families based in camps or resettling in villages close to camps, caregivers could commute to the casual labour site or village lands daily for work, returning in the evening. Families whose villages were far away could not move back and forth daily and left camps for extended periods of time, some reporting weeks or even months. This meant having no time to teach, interact with, or monitor their children. While participants expressed the vulnerability of this situation many talked of having no alternatives. Early resettlement was lamented as being a very difficult time for children who were exposed to a myriad of factors acknowledged to hinder health, development, and well-being.

… Because of this poverty the child cannot get enough food, nor enough clothes, nor enough teaching because the parent goes about looking for avenues to earn a living; there is now no time for guiding the child. —*28-year-old male Local Council Leader in return to village. *

Participants revealed a hierarchy of preferred care options for young children who had to be left behind; a supervising adult such as a grandparent or neighbour as a first preference, followed by an older child in charge, and lastly, children left amongst their peer groups. Sadly, reports suggested that many of the elders who would traditionally have provided supervision while parents were out cultivating had either died during war or were themselves forced to go out and work for survival. Additionally, many examples were observed of grandparents who had once again assumed the role of primary caregivers because parents had died, been abducted, or left their children.

… In the past we spent a lot of time with our grandchildren, but now, you cannot spend much time with them because you have to look for means of survival. These days we the dayo (grandmothers), we go to the garden because we lost our sons who would have gone to do the work we are doing now and the gardens are far; you go in the morning, leaving when all you have done is sweep the compound and you just leave water for the child to use to wash his or her face. You go away and come back in the evening now to begin preparing food for them. Your grandchild may spend lunch without food but in the past, we could feed them at least two to three times in a day. We now do not have time to bathe our grandchildren. We do not have time to tell them stories. The time you are supposed to be telling them stories, you may be too tired to even talk. So, I see that our role has changed just because we are not spending much time with our grandchildren, we do not have time to teach them, we do not have time to cook food for them, we do not have time to play with them because we get involved in other work like casual labour, petty trade, and digging in the garden. *FGD with elder women. *

#### 3.3.2. The Vulnerability of Children without Parental Care and Provisions

In Acholi culture, children had always assumed a care-giving and stimulation role for other children, so child-to-child caretaking during resettlement was not new. It was a time-proven practice that served to meet many of the stimulation and care needs of children within the folds of the extended family. However, what was acknowledged to make transition a uniquely vulnerable period was that children were frequently reported to be without adults in the vicinity to supervise and assist the child caretakers. In one remote camp, the team observed dozens of children of all ages without supervising adults. Additionally, people spoke of children being left without food or money to sustain themselves while parents were away working. The following excerpt from FDG with siblings who remained in a camp illustrates some of the serious issues faced by children when parents were gone to the village for prolonged periods.

Participant 2. There is always no one; they always go away all to the garden and you find very few adults within the camp and I may not be knowing them and cannot go to them for help even when something happens; even when a child is sick…… Night hours are a nightmare to us.

Participant 3. We suffer at night. They (adult intruders) carry away our food and we hear them saying “your mother is not around, your mother has gone to the village.”

Participants 1. They always see and notice that our parents have left for the village.

Participants 4. They may even know you and your parents.

Participants 2. At times we do not know the people who come to steal; we wake up in the morning not knowing who has taken our food, but at times we know the people. *FGD with 4 half-siblings between the ages of 9 and 13 living in camp with younger siblings while parents return to the village to work and rebuild. *

A situation of even greater vulnerability occurred when young children were left amongst their peer groups (i.e., groups of children 5 years and below). This would happen if older children were in school and therefore unavailable for care or if there were no older children or supervising adults available in the family. This practice was observed in all camps and was noted by all categories of participants. 

An informal interview with an employee of UNICEF indicated that “children deprived of parental care” was a major issue experienced during transition. The Child Protection Committee reports indicated that this issue composed 23% of reported cases in 2009. The following excerpt is from an FGD with the Child Protection Committee members:

Participant 4. Ah what I have seen happening here these days which is very bad in Pabo here, most people returning home, they leave children behind without food and you find the children moving aimlessly in the camp, they move near restaurants, you find them picking up discarded food and discarded millet bread, the ones which people have eaten and left….

Participant 6. Just yesterday I saw some two parents leaving their young children behind, children of 1 year and 2 years and even 3 years, they leave them home for the whole day, they go to their gardens for long hours, come back late in the evening and you will find the children hungry the whole day or not even breast fed….

Participant 1. There are those who do not have lapidi (child babysitter). You see, for us here once a child lacks a lapidi that child will always suffer the moment the parents leave for the garden. Such a child will try to survive on his or her own and will not know how to differentiate that this thing is good or bad.

As the previous quote illustrates, when young children were left without adult supervision and provisions, participants reported a number of very serious outcomes. The following outcomes which appeared in transcripts are listed in order of frequency of mention: hunger (and subsequent malnutrition); falling seriously ill or being injured (falls, burns, bites, beatings) with nobody to help; suffering and distress experienced without comfort provided; missing out on teaching, guidance; picking up bad behaviours such as stealing, fighting; wandering around or loitering aimlessly and unengaged; and being subject to predation by others in camp, such as coercion, rape, theft. Acholi caregivers placed great value in their children and as many of the quotes have demonstrated, there were high levels of knowledge regarding risks and outcomes if children's needs were not met as well as the factors that would offer protection for their children's futures. Yet the circumstances in postconflict transition rendered many unable to change their children's environments and experiences.

… With children if they miss all that we have been telling you of (their needs), in future nothing will come out of them. There is nothing that they will do that will be progressive. If we cannot help our children now, and we say that we shall help them maybe in two years from now, we shall be wasting our resources because they will not be much of a help to us…. Our children will not be able to study, we shall not have a good generation. If we begin taking care of our children well now and we then take them to school, they will complete their education well, they will be able to support us and even support other great grandchildren. But right now we have spoiled what would have been good for the future of our children. Our children will grow up to be people who always commit crimes. Our children, because they are not getting enough food and sicknesses disturb them a lot, in the future, we shall have children who all have mental problems. —*Traditional birth attendant FGD participant, Pabo camp. *

#### 3.3.3. Social Protection Offered by the Village

When families resettled fully in their villages with their children, they began to report lessening degrees of vulnerability. In villages, even when parents were working in the gardens, they or another adult was generally within earshot and could hear children's cries or children could go to the adults if needed. Homesteads were spaced far apart with only vast expanses of agricultural land and bush in between so children would remain near the home where they generally had access to food, water, shade, and wide open, controlled spaces for play.

I have realized some changes at least in coming back home in the life of my children because the condition in which they were when in the camp was not good. The camp used to be so oppressive they could hardly play well. There was no particular playground or play space that they could find in the camp, they were not free. But now that they have come back to the village, they play anywhere and they are very free, they run over to the other tree, then come back to the other side and at least they have rested. They are even quite free (the wind is blowing around them). Even me, as a mother, I am feeling so free. I do not have to keep on controlling my child so closely, protecting my child “oh, I have to protect my child from so and so, or perhaps my child might do something bad in some place or to someone's property who is not around”. —*Case study: 27 y.o. married woman, caring for 7 children. Recently transferred to the village from a nearby camp*.

The data showed a positive trend in social-cultural support restoring across time when families resettled successfully in the village. After so long in camps where parents spoke of having very little control for ensuring positive nurturing environments for their children, return was a most welcome restoration of the highly valued social cultural ways of the past. This included restoration of practices such as the nightly wangoo, or family campfire, where family would come to unite, plan out how to meet needs, share songs and stories, and teach children.

### 3.4. The Regional Resource and Service Environment of Children after Conflict

This section will illustrate themes relating to the regional resource environment of postconflict transition as it relates to young children. The major subthemes are (1) lack of services in return sites and (2) problems with targeting and implementation of services.

#### 3.4.1. Lack of Services in Return Sites

As was covered above, participant reports suggested that the majority of services provided to children during war and displacement by nongovernmental and international organizations ceased to operate or were scaled down significantly when war ended and resettlement began.

… Now, when people are returning to their original homes, there should be programs which should be targeting them when they are going back to their original sites, but at the moment they are not, there are no programs which are actually targeting people who are returning to their original sites, most especially when you talk about the children… —*Government official, Amuru District. *

While participant observation revealed that there were a range of interventions operated by development partners and government during the postconflict period such as agricultural support/training programs, assistance with repatriation and/or food aid for “extremely vulnerable individuals” (people with disabilities, the elderly, persons who are HIV positive), hospital-based nutritional supplementation for children who attended with serious undernutrition (when supplements were available), and building of infrastructure such as health centres and schools, many of these services were insufficient in scale or too centralized in location to meet the needs of the dispersing population. Furthermore, while many programs benefited children indirectly through bolstering the family unit, few programs targeted young children directly. For instance, in the Pabo camp some participants recalled approximately 5 early childhood centres that had existed during war; however, all but two had been abandoned and fallen into disrepair during the postconflict transition—a time when they would have been highly beneficial. The two that were confirmed to be in existence either charged high fees that were unattainable to most or targeted specific populations such as children with disability.

As one employee of an international NGO reported, there was a significant funding gap after conflict, the conflict-oriented funding had been withdrawn, and development funding was insufficient to replace it. This was a point of resentment and frustration clearly for leaders and development workers, but even more so for many caregiver participants. Upon return to villages (particularly remote villages), they lost or had substantively reduced access to services and infrastructure for their children. These were services that had come to be highly valued during camp living like health care, clean water, and education. Some villages reported a lack of markets that made it difficult to access needed staples like soap, salt, sugar, and to sell foods that they had grown in order to get resources to support children.

… The lack of school and then also missing a big therapeutic/supplementary feeding centre to encourage the brain and thinking of our children. So ignorance is going to be the result in the lives of our children…. Because there is no hospital, most of our children will now not live. The result will now be many graves filling up the whole place. People say that there is change with the dawning of the day, but it looks like with us, the dawning of the day only brings us problems every day. —*30-year-old rwot kweri (traditional chief) returned to village*.

… What is causing life to be difficult now that people are returning and the war is ended is that where people are returning to there is no proper water, no hospital, no schools…. That means it is not easy to keep good health among the people. —*25-year-old father of three, husband to two women, living in small transition camp; ancestral land is far away and is under dispute. *

#### 3.4.2. Problems with Targeting and Implementation of Services

Services that were being offered for children were noted by some to be a poor match for the most pressing needs. For example, nutritional education being provided during drought when children did not have food to eat; or health centres open only during daytime hours. Some services designed to reach children at the village level such as the village health teams (VHTs) who were to monitor, provide health education, and dispense basic drugs such as malaria treatment, were spoken of as having started out strong, but then faltered in effectiveness due to a lack of resources such as drugs and support, compensation of workers, and monitoring.

… It (VHT) has reduced the strength and level of sicknesses that disturb children—it had actually gone down—but beginning from the end of the past year when the medicines were finished, that is when sicknesses began to disturb children again so much. Also in this place the death rate has been going higher. —*25-year-old male village health team member and Rwot Kweri (traditional chief). *

This pattern of faltering services was reported repeatedly by community leaders, both government and frontline workers. They spoke of the deficiency of monitoring, lack of sustained support, and limitations to the evaluation of implemented programs. While organizations were strong on initiating, participants felt follow-through was often poor and they were neglected in communities as the program that had offered so much promise for their children petered out. This leads to expressions of both disappointment and a consequent lack of trust in the promises made for their children.

… When these (early childhood care) centres are started by these development partners, people have very high expectations. So people started sending their children to these centres because the caregivers have been promised they will pay just a certain amount of shillings. But now when conflict ended people (implementers) began just to desert the centres … and that is why you see them dying out because the NGO started them and when their contract expires, we have no support for that centre. *Amuru District official. *

Some participants recounted maintaining their homes in the camps for longer periods or leaving their children behind because of proximity to services such as schools and health centres which were not available in return sites.

The one program consistently reported to reach children, even those in distant rural locales, was immunization services. Immunizations were provided through the cooperation of government agencies, international organizations such as UNICEF and NGOs on an outreach basis. Community leaders would mobilize their people to gather on the expected dates to receive services and reports suggested high levels of satisfaction.

## 4. Discussion

In the aftermath of a vicious 20-year war in northern Uganda, caregivers and communities struggled to rebuild their lives and ensure their children's survival. In the recovery from more than a decade of displacement, the major resource available to facilitate their transition home was hard, prolonged labour to restore agricultural lands, rebuild resources and homesteads. Caregivers engaged in this work and strove to get their children out of camps so they could return to traditional childrearing practices. They longed for times when resources such as food and livestock were available, the places where children lived and played were controlled, and the family surrounded and nurtured young children. But through the difficult process of transition, the reality they reported was that the environments of early childhood were not supportive, safe, or nurturing.

The aftermath of war brought continued vulnerability to the youngest members of society whose rapidly developing brains and bodies were laying down the foundations of their future well-being. Young children experienced numerous risks to their health and development, risks that previous research has documented as threatening to long-term developmental outcomes [6, 12, 40]. These risks included malnutrition, poor health, and survival-based neglect in the form of reduced care, supervision, teaching, and stimulation. As risks accumulated, so did the threat to cognitive, social-emotional, and/or physical well-being; yet, as noted by Wessels and Edgerton [41], accumulation is typical of too many children both during and after war [42]. Furthermore, while caregivers and community leaders showed high levels of awareness about the risks to young children, their poverty, lost social support, and necessary focus on survival often prevented mitigation. Support and assistance was also not forthcoming from external sources. The findings of this study suggested that, when emergency focused interventions were withdrawn after conflict, services remained largely centralized and were not scaled sufficiently to address the needs of young children. Furthermore, some interventions that did exist were felt to be poorly targeted or lacked monitoring, evaluation, and sustainability.

There are approximately 26 million internally displaced people globally, people who remain within their own countries but who are forced to leave their homes due to conflict, violations of human rights, and/or generalized violence [41]. The African continent is the most impacted by internal displacement with an estimated 11.6 million people affected, approximately half of whom are children [41]. These children are away from their home environments and social supports that provide routine and nurture during the most critical stages of their development. Moreover, as the narratives of Acholi caregivers in northern Uganda bring to light, when the emergency phase concludes, the vulnerabilities faced by young children can persist, particularly when displacement has been prolonged. There is vast and growing evidence documenting the critical importance of the early years, which has led to policy and interventions that strive to mitigate risks to children and meet the unique needs of early childhood. International bodies such as the United Nations, the World Bank, and the World Health Organization have called upon governments to increase attention, training, and spending for early childhood development (ECD) in order to create holistic, accessible, and culturally appropriate programs that involve families and communities and are based on partnerships [43]. Unlike that in the past, ECD now has a significant presence in international policy with charters and declarations such as the World Declaration for Education for All (UNESCO, 1990), the Convention on the Rights of the Child (UN, 1989), The African Charter on the Rights and Welfare of Children ACRWC (1990) [44], and the Organization for African Unity's: “Africa Common Position: Africa Fit for Children” [45]. Today ECD is also included in two of the major global targets for development—the Millennium Development Goals (MDGs) and Education for All (EFA) [46]. Nonetheless, the calls for action are insufficient if they cannot or do not address those difficult to reach, the children who are exposed to the most difficult environments, and the greatest levels of risk such as those highlighted in this research and supported by others in the field [16–19].

As the report on early childhood from the World Health Organization's Commission on Social Determinants suggests, families need support and sustained commitment from regional, national, and international communities to provide nurturing environments for children and preserve their developmental potential [2]. Yet participants in this study indicated that there is a continued need to strive to develop and/or effectively implement policies and practice guidelines for government, international organizations, and nongovernmental organizations that focus on protecting young children and supporting their healthy development in war and displacement. Such initiatives should include obligations of continued context appropriate support beyond the period of conflict in recognition of dependency, expectation, and need created within the population. While the challenges of providing such support are numerous [42], the potential long-term risks of not doing so, for both child and society, are too great to forego.

In northern Uganda, risks to young children in postconflict resettlement could have been mitigated to a much higher level. Nonetheless, the experiences of these children should inform better approaches in the future. Targeting is required at both the macro- and microlevels to rebuild communities and environments that support and enable children and directly meet the needs of individual children [42]. For instance, while policies exist to address the separation of children from caregivers in war zones, this does not acknowledge the separation that occurs when caregivers must choose to leave children for the sake of rebuilding livelihood following conflict. Yet, the outcomes for young children in these situations can be the same—long periods of time without the protection, care, and attention of a primary caregiver and exposure to a myriad of risks from the social and physical environments. Findings of this study suggest a need for increased efforts to implement culturally appropriate childcare solutions following conflict led by the ideas and practices of local people to facilitate the well-being of children throughout the transition process. Potential solutions may be found in the poverty-reduction strategies already being employed at the grassroots village level such as in cooperatives of rotating caregivers, the trade of goods, and/or labour for childcare services.

Another example of an area of need, which the participants of this study reported to be inadequately addressed was nutritional vulnerability during postconflict transition. While there were categories of “extremely vulnerable individuals” (EVIs) targeted for extra food aid in northern Uganda, reports and observation suggested that young children were not amongst them. Acknowledging that continued dependence on food aid is not a desirable outcome of programming during resettlement, it also must be acknowledged that young children's brains and bodies are particularly vulnerable to nutritional deprivation and that long-term outcomes can be serious [12, 40]. Targeting young children and breast-feeding and pregnant mothers as categories of “EVI” deserves further consideration.

On a more macrolevel, findings suggested a need to proactively implement innovative ways of reaching dispersing paediatric populations in postconflict resettlement. Participants indicated a need for services such as accessible healthcare, clean water, and early childhood education interventions. Grassroots research is required to guide the planning and monitoring of such services so the realities and most pressing needs of those who are dispersed and hard to reach drive the solutions. When commitments are made and services are implemented that reach communities, greater attention and action is required to sustain programs and/or find realistic solutions for transferring them to local government or communities. The participants of this study expressed great frustration and disappointment over having programs introduced only to see them fail shortly thereafter due to a lack of monitoring and ongoing support. Money invested in services that fail, or which reach only limited, centralized populations and miss those with the greatest need, could be better directed. In northern Uganda, services such as immunization outreach were reporting success, yet such remote outreaches were perhaps underutilized as a means of providing additional services that address health needs. Interventions such as the Child Protection Committees or village health teams working at the community level offered tremendous promise in meeting young children's needs—members were highly knowledgeable about the risks being faced by young children within their communities. However, if no follow-up support is given to address the risk identified by these teams, frustration ensues, work diminishes and children lose this resource. Implementers need monitoring and evaluation research that gathers evidence as to whether or not interventions are succeeding, as well as why or why not.

This exploratory study shed light on factors threatening the developmental potential of young children in postconflict northern Uganda. It offers a starting point on which further study could be based; however, some limitations are noted. It did not include all districts in the north nor urban dwellers; the extent to which these people face similar issues is unknown. While case study methodology offered some longitudinal perspective, much of the data was cross-sectional, capturing a moment in time. Long-term followup would offer greater perspective on outcomes for children. Two researchers conducted participant observations, and their positioning will differ from other researchers. Nonetheless, their audit trail and debriefing with other research team members offers some assurance of rigor in the analysis and interpretation of findings. There may have been recall bias in the retrospective accounts, or participants may have sought to tell the researchers what they wanted to hear or present a situation to elicit service delivery. However, relatively large sample of participants, range of data sources, and volume of data help to mediate any systematic respondent bias.

## 5. Conclusions

War and displacement have profound impacts on young children and their families. Families who are rebuilding their livelihoods after such devastation need support to move beyond a survival focus and to recreate nurturing environments for their children. Regional, national, and international bodies have acknowledged the critical importance of early childhood and laid the policy and resource foundations to meet needs, but, as is shown in this study, more must be done to tangibly assist those living in the most difficult environments who are the hardest to reach and the most vulnerable. Failure to do so jeopardizes the future of a healthy prosperous society. Societies impacted by war desperately need to preserve the human capital that will ensure that the future is better than their past. As the 2011 World Development Report on Conflict, Security and Development [47] states:

No low-income fragile or conflict-affected country has yet achieved a single MDG (Millennium Development Goal). People in fragile and conflict-affected states are more than twice as likely to be undernourished as those in other developing countries, more than three times as likely to be unable to send their children to school, twice as likely to see their children die before age five, and more than twice as likely to lack clean water. On average, a country that experienced major violence over the period from 1981 to 2005 has a poverty rate 21 percentage points higher than a country that saw no violence… (p. 5).

Health, peace, and development cannot exist without a healthy thriving human population, and a thriving human population cannot exist without healthy, peaceful, thriving children.

## Figures and Tables

**Table 1:** Description of research sites.

*Pabo subcounty*	Pabo camp had been one of the largest and most overcrowded camps during the war. Numerous emergency relief organizations had built up infrastructure in the area. As such, it was a relatively well-resourced and accessible site located on a main road with two health centres, schools from nursery to secondary, markets, and other resources. The majority of participants from this site were residents of the Pabo camp and/or were in early phases of resettlement planning their return or moving back and forth between camp and villages. Half of the caregiver participants became case studies and those who resettled were followed back to their villages of resettlement in the subcounty.
*Amuru subcounty*	Site was very remote, difficult to access, and underresourced. The team travelled by car, motorbike, and hiking on foot. All but one of the water points in the camp were broken, the nearest health centre was approximately 11 km away and it was only open during weekday daytime hours, the road was badly rutted with potholes filled with mud and water, and the primary school was a mud and grass-thatched structure built and supported by the community. Participants were either from the Omee II decongestion/transition camp (small camp established later in the war to move people closer to their land for subsistence and to decongest larger camps) or the surrounding villages. The majority of participants were either actively in the process of resettlement (moving back and forth to camp) or had recently resettled.
*Anaka subcounty*	Accessibility and resources/services were variable; that is, villages closer to the main centres tended to have access to health care, education, and water points whereas those farther away from the centres were more like the Omee II with very limited or inaccessible resources. The third site consisted of 8 parishes (villages and camps) in the Anaka subcounty in order to capture people who had been resettled for varying durations and reach saturation on emerging themes.

**Table 2:** Demographics of participants.

	Leaders	Primary female caregivers	Fathers	Elders	Sibling caregivers
Age range	20 to 68 years	16 to 72 years (included grandmothers caring for orphans)	22 to 64 years	42 to 92 years	4 to 15 years
Sex	28 male 23 female	—	—	8 male 14 female	6 male 23 female
Highest level of education achieved	No formal education to graduate level training	18 no formal education 17 primary	23 primary 1 senior 1 postsecondary diploma	15 none 5 primary 2 senior	5 none (some too young) 24 primary (some not currently in school due to poverty)
Livelihood	Leadership positions described above and/or farming (particularly for LCs and traditional leaders)	Farming either in own land or for others for food or money and/or small-scale trade/ business	Farmers	Farming, small business, receiving aid, or supported by family	*n*/*a*
Marital status	*n*/*a*	23 married or living together 12 separated or widowed	All married or living together (1 to 4 wives)	10 married 11 widowed 1 separated	*n*/*a*
Number of children range	*n*/*a*	1 to 8 children (9 were caring for orphans)	3 to 14 (including biological and orphans)	4 to 11 including biological and orphans	Most caring for 1 to 2 charges
Current residence	*n*/*a*	14 in camp 3 living both in camp and in village 16 in village	10 in camp 5 in both camp and village 10 in village	8 in camp 14 village	14 in camp 15 village (most were born in camps or came in early infancy)

**Table 3:** Examples of questions guiding interviews and focus groups.

Postwar living arrangements	
(i) If still in the camp, explain why?:	
Tell me about the impact of remaining in the camp for your children:	
(ii) If in the village, tell me about your return to the village:	
Tell me about the impact of returning to the village for your children:	

Current service environment	
(i) Describe the services that are currently available for young children:	
(ii) Who are the existing or potential leaders who are (or could) address the needs of young children in your community?	

Nutrition	
(i) Describe what you should feed [NAME]:	
(ii) Is there anything that makes it difficult or stops you from feeding [NAME] these foods? (probe: environment factors, social factors, number of times issue is experienced)	
(iii) Is there anything that helps you feed [NAME] these foods?	
(iv) Describe what happens when you cannot feed her/him the foods you describe:	

Health	
(i) Describe what [NAME] needs to be healthy:	
(ii) Describe a safe and happy environment for a young child:	
(iii) In the last two weeks has [NAME] been ill? (Probe illness and treatment.)	

Caretaking practices	
(i) Who spends the most time with young children? (Probe who takes care when caregiver is away or busy.)	
(ii) How are young children supervised?	

Developmental stimulation	
(i) Describe what children learn in the (1st, 2nd, 3rd year of life-ask according to age):	
(ii) How do they learn each of the skills you mentioned?	
(iii) What does play look like for a child this age? (Probes: whom do they play with? Show me what he/she plays with.)	

Questions about young children in war and displacement	
(i) Describe what was different about raising young children before war when people lived in their villages:	
(ii) Did this change during war? (describe)	

Risks/barriers and enhancers/facilitators	
(i) What is bad for young children? (i.e., what could prevent them from being as successful as possible?)	
(ii) What should be done to make sure [NAME] and all young children have the chance to become successful adults?	

Future outcomes:	
(i) What do you want for [NAME]'s future? (Probes: valued outcomes, measures of success, desirable character traits.)	
